# A Unique Bilateral Anatomical Variation of the Transverse Facial Artery: A Case Report

**DOI:** 10.7759/cureus.30511

**Published:** 2022-10-20

**Authors:** Carley Olson, Yun Tan, Meadow Campbell

**Affiliations:** 1 Department of Surgery, Saint Louis University School of Medicine, St. Louis, USA

**Keywords:** anatomical variation, maxillofacial surgery, carotid termination, transverse facial artery, facial artery

## Abstract

During routine dissection of the superficial face in an 81-year-old male cadaver, an unusually large caliber transverse facial artery (TFA) was observed bilaterally. Further dissection revealed the presence of a hypoplastic facial artery (FA) that passed deep to depressor anguli oris and gave off the inferior labial artery. Bilaterally, the TFA gave rise to the superior labial artery, lateral nasal artery, and ended as the angular artery. Anastomosis of the branches of the TFA, FA, and infraorbital artery was noticed at the buccal area. To our knowledge, there are no previous reports of this anatomical variation. Documentation of variations in the arterial supply of the face will be helpful in further minimizing complications during facial surgery and cosmetic procedures.

## Introduction

The vasculature of the face is essential in maintaining healthy tissues and is composed of numerous anastomoses. The FA is known to be a major artery supplying blood to the muscles and skin of the face [1]. It arises from the anterior aspect of the external carotid artery, courses upward deep to the submandibular gland, and wraps around the mandible to pass on to the face [1]. It takes a tortuous route along the course of the face to allow for movement and contraction of the muscles of mastication and facial expression [2]. Once on the face, the FA typically gives off four main branches: the inferior labial artery, superior labial artery, lateral nasal branch, and angular artery.

The superior and inferior labial arteries typically branch from the FA around the intersection of the vermillion borders of the upper and lower lip. The superior and inferior labial arteries traverse between the mucosa and orbicularis oris muscle and anastomose with the superior and inferior labial arteries of the contralateral side. The inferior labial artery typically branches at the angle of the mouth, courses anteriorly to run beneath depressor anguli oris, then pierces the orbicularis oris muscle [3].

The superior labial artery travels medially towards the philtrum to provide blood supply to the upper lip as well as contributes branches to supply the nasal septum [3]. The lateral nasal artery is the remaining branch of the facial artery (FA) after it gives off the superior labial artery near the oral commissure. As the lateral nasal artery turns anteriorly along the nasal ala, the angular artery continues superiorly along the lateral aspect of the external nose [3].

The FA is known to commonly exhibit variations in its origin, course, termination, and branching pattern [4]. In an effort to evaluate the different types of branching patterns of the FA, Hong et al. classified four different types of FAs using conventional angiography. Type 1 describes a FA that has the angular artery beyond the midline of the orbit as its terminal branch [5]. Type 2 has the lateral nasal artery as its terminal branch, Type 3 has the superior labial artery as its terminal branch, and Type 4, which the FA only has the inferior labial branch [5].

The TFA is a branch of the superficial temporal artery that provides blood supply to the lateral aspect of the face. It is usually a small vessel, resting on masseter muscle, running transversely between the lower border of the zygomatic arch and parotid duct and ending in the buccal area [6].

This case report adds to the literature regarding variations in the origins of vasculature within the superficial face. To our knowledge, there have been no previous reports about an enlarged TFA with a hypoplastic FA bilaterally. We believe that the coexistence of these unique variations is of high clinical significance and should be considered during facial reconstruction and cosmetic plastic surgeries in this region.

## Case presentation

This 81-year-old male donor was received through the Saint Louis University (SLU) Gift of Body Program of the Center for Anatomical Science and Education (CASE). Appropriate consent and approval were obtained from the family of the donor. The donor’s self-reported medical history includes Alzheimer’s disease, Factor V Leiden, coronary artery disease, COPD, deep venous thrombosis, and hypertension. The authors state that every effort was made to follow all local and international ethical guidelines and laws that pertain to the use of human cadaveric donors in anatomical research [7].

**Figure 1:**
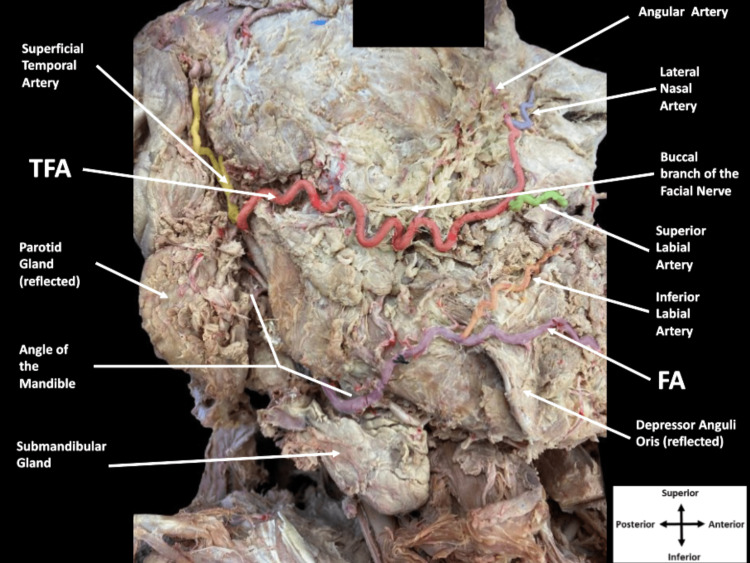
Dissection of the right side of the superficial face. The right transverse facial artery (TFA) highlighted in red branched from the superficial temporal artery (yellow) deep to the parotid gland, coursed medially across the face alongside the buccal branch of the facial nerve and gave off the superior labial artery (green) and lateral nasal artery (blue) then continued as the angular artery (pink). The facial artery (FA) highlighted in purple emerged inferior to the angle of the mandible and coursed deep to the depressor anguli oris muscle after giving off the inferior labial artery (orange).

Upon removal of the skin and superficial fascia during routine dissection of the superficial face, an unusually large caliber TFA branching from the superficial temporal artery was discovered. Bilaterally, the large TFA coursed medially taking a tortuous route superficial to the masseter, zygomaticus major, and zygomaticus minor muscles alongside the buccal branch of the facial nerve. The vessel reached the upper aspect of the angle of the mouth before giving off the superior labial artery (Figure 1).

The superior labial artery continued medially in its expected manner to supply blood to the upper lip and nasal septum then anastomosed near the midline with the contralateral superior labial artery. After giving off the superior labial artery, the TFA traveled superiorly in the nasolabial fold toward the ala of the nose to give off the lateral nasal artery, which took its usual path to supply blood to the ala of the nose. The vessel then continued superiorly on the lateral aspect of the nose as the angular artery before terminating near the medial angle of the orbit.

**Figure 2:**
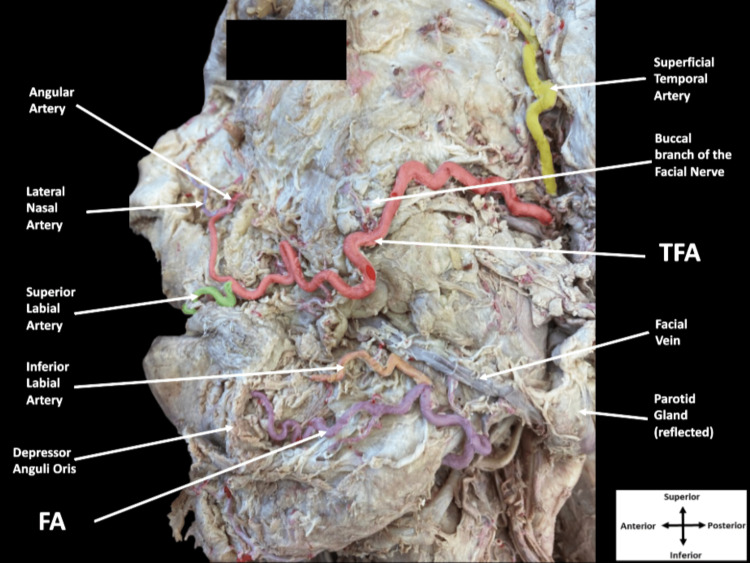
Dissection of the left side of the superficial face. The transverse facial artery (TFA) in red branched from the superficial temporal artery (yellow) and branched off the superior labial artery (green) and the lateral nasal artery (blue) before continuing as the angular artery (pink). The facial artery (FA) in purple emerged inferior to the mandible, coursed medially to give off the inferior labial artery (orange), and passed deep to depressor anguli oris supplying the infralabial muscles.

Further dissection revealed the presence of a hypoplastic FA originating from the external carotid artery. The FA took its expected course as it traveled deep to the submandibular gland, emerged inferior to the mandible, and passed onto the face (Figure 2).

Once on the face, the FA coursed superomedially and gave off the inferior labial artery. Both vessels continued medially and passed deep to the depressor anguli oris muscle. The inferior labial artery traveled superomedially toward the inferior aspect of the mouth to provide blood supply to the lower lip as expected. The FA coursed medially before terminating to supply the infralabial muscles and forming small anastomoses with the mental artery. Based on the study performed by Hong et al., the FA in this case report would be classified as a Type 4 [5]. Anastomoses between the TFA, FA, and infraorbital arteries as well as between the inferior labial and mental arteries were observed.

## Discussion

The TFA plays an important role in lateral facial vascularization, and there are few published papers describing variations of the TFA. A study by Koziej et al. found that the TFA was present in 192 out of 200 (96%) cases. Of the cases documented, only one (0.5%) was associated with a TFA that was dominant over a hypoplastic FA [6]. The presence of a bilateral TFA dominant over a hypoplastic FA was not observed. In our study involving 90 hemi-faces, only two (2.2%) hemi-faces demonstrated a TFA that was dominant over a hypoplastic FA. Both hemi-faces belonged to the male cadaver of this study. Another case study describes a similar finding in which a TFA compensated for a hypoplastic FA bilaterally [8]. However, the authors observed that once the FA reached the superficial face, it bifurcated into two atypical branches, terminating in the buccinator muscle. The TFA supplied compensatory blood flow to the superior labial, lateral nasal artery, and angular artery. The study does not mention the presence of an inferior labial artery. Another study performed by Tubbs et al. Reported a giant left TFA compensating for the complete absence of the ipsilateral FA; however, this was a unilateral anomaly [9].

Although there have been many previous studies performed to better understand the prevalence of various courses of the FA, the results significantly differ, and a consensus has yet to be established [10]. However, termination of the FA as the inferior labial artery is one of the least prevalent courses. For example, one study using angiography was performed by Hong et al. to evaluate 284 cases of FAs in 198 adults. They found that in 104 cases (36.6%) the FA terminated as the angular branch, 138 cases (48.6%) terminated as the lateral nasal, 24 cases (8.5%) terminated as the superior labial, and 18 cases (6.3%) only had the inferior labial branch [5]. Mitz et al. reported a study of 50 cadaveric specimens with 78% terminating as a lateral nasal artery, 10% as a superior labial artery, 4% as an angular artery, and 8% as an inferior labial artery [11]. Koh et al. studied 201 specimens to classify the branching pattern of the FA and found that 20% of the FAs terminated as the angular artery, 48% as lateral nasal, 17% as alar, 10% as superior labial, 3% as inferior labial, and 2% took an abortive course [12]. Though numerous studies have found many variations involving the FA, we have not encountered a case involving the coexistence of an enlarged TFA from which the superior labial, lateral nasal, and angular arteries originated and compensated for a hypoplastic FA bilaterally as observed in our case study.

The FA and TFA are derived from the external carotid artery, which develops from the third aortic arch of the aortic sac during the fourth and fifth weeks of embryological development [13]. According to an analysis performed by Bruë et al., the anatomical variations observed are determined by regional hemodynamic balances. These observations may influence present concepts concerning the hemodynamics of arteriovenous malformations of the face and may serve as an explanation for the variations observed in our study [14].

The novel variation described by this paper is of high clinical significance and should be accounted for during surgical procedures on the face such as maxillofacial, aesthetic, and plastic surgery. Lip reconstructions such as the Abbe flap, Estlander Flap, Gillies Fan Flap, and McGregor Flap procedures involve surgical manipulation of one of the major FA branches: the superior labial artery or inferior labial artery [15,16]. For this reason, awareness of the possibility that the superior labial artery, lateral nasal artery, and angular artery can branch from the TFA bilaterally is relevant to these lip reconstructions.

In aesthetic surgery, a facelift flap is a common procedure, and TFA perforators are one of the most important arterial sources for this flap [17, 18]. Damage to the TFA can cause a reduction of blood supply to the facelift flap that may lead to surgical failure. Moreover, in a case in which the TFA gives off the superior labial artery bilaterally as described in this study, maintaining adequate blood supply to the upper lip and lateral nose may complicate this facelift flap procedure. The most common complication of a facelift flap is a hematoma, which can endanger the survival of the flap. The most significant cause of this complication is direct TFA transection [19]. In this case, not only would the facelift flap be vulnerable to failure, but blood supply to the upper lip and lateral nose may be compromised.

The TFA and FA are also present in the area of interest of trauma surgeons as injury to the face caused during a traffic accident or explosive accident is possible [19]. Surgeons performing procedures near the course of the TFA should be aware of the possibility of an enlarged TFA and its unusual branches as described in this study to avoid negative patient outcomes.

## Conclusions

During routine dissection of the superficial face, a unique variation involving the TFA and FA was observed. To the best of our knowledge, there have been no documented cases of the coexistence of a bilateral TFA that branches into the superior labial artery, lateral nasal artery, and angular artery, along with a hypoplastic FA that contributes to the inferior labial artery. This case report adds to the literature regarding variations in the origins of vasculature within the superficial face. We believe that these unique variations are of high clinical significance and should be considered during facial trauma, facial reconstruction, and cosmetic plastic surgeries in this region to prevent complications.
